# Destroy and Exploit: Catalyzed Removal of Hydroperoxides from the Endoplasmic Reticulum

**DOI:** 10.1155/2013/180906

**Published:** 2013-10-24

**Authors:** Thomas Ramming, Christian Appenzeller-Herzog

**Affiliations:** Division of Molecular and Systems Toxicology, Department of Pharmaceutical Sciences, University of Basel, Klingelbergstr. 50, 4056 Basel, Switzerland

## Abstract

Peroxidases are enzymes that reduce hydroperoxide substrates. In many cases, hydroperoxide reduction is coupled to the formation of a disulfide bond, which is transferred onto specific acceptor molecules, the so-called reducing substrates. As such, peroxidases control the spatiotemporal distribution of diffusible second messengers such as hydrogen peroxide (H_2_O_2_) and generate new disulfides. Members of two families of peroxidases, peroxiredoxins (Prxs) and glutathione peroxidases (GPxs), reside in different subcellular compartments or are secreted from cells. This review discusses the properties and physiological roles of PrxIV, GPx7, and GPx8 in the endoplasmic reticulum (ER) of higher eukaryotic cells where H_2_O_2_ and—possibly—lipid hydroperoxides are regularly produced. Different peroxide sources and reducing substrates for ER peroxidases are critically evaluated. Peroxidase-catalyzed detoxification of hydroperoxides coupled to the productive use of disulfides, for instance, in the ER-associated process of oxidative protein folding, appears to emerge as a common theme. Nonetheless, *in vitro* and *in vivo* studies have demonstrated that individual peroxidases serve specific, nonoverlapping roles in ER physiology.

## 1. Introduction

Hydrogen peroxide (H_2_O_2_) is an intracellular metabolite, which serves important roles as a second messenger in redox signaling [1]. However, since elevated levels of H_2_O_2_ (and of other reactive oxygen species, ROS) can damage proteins, nucleic acids, and lipids by peroxidation, temporal and spatial limitation of H_2_O_2_ levels is critically important. Thus, half-life and spatial distribution of H_2_O_2_ in the cell are tightly regulated by nonenzymatic antioxidants as well as by specific scavenging enzymes, including the so-called peroxidases of the peroxiredoxin (Prx) or glutathione peroxidase (GPx) families [2]. Prx and GPx isoforms reside in different subcellular compartments where they catalyze the reduction of H_2_O_2_ to H_2_O [2]. The most relevant producers of intracellular ROS/H_2_O_2_ are the transmembrane enzyme complexes of the nicotinamide adenine dinucleotide oxidase (NOX) family, various enzymes and the respiratory chain in mitochondria, peroxisomal enzymes, and sulfhydryl oxidases in the endoplasmic reticulum (ER) [3–7]. Due to the presence of specific aquaporin channels in cellular membranes, the local diffusion of H_2_O_2_ is usually not restricted by organelle boundaries [8, 9]. 

There are a total of six isoforms of Prx in mammals, all of which form distinct types of antiparallel homooligomers [10]. H_2_O_2_-mediated oxidation of the active site peroxidatic cysteine (C_P_) to a cysteine sulfenic acid is a common feature of Prxs. However, only so-called 2-Cys Prxs possess a resolving cysteine (C_R_), which attacks the C_P_ sulfenic acid, leading to the formation of a C_R_–C_P_ disulfide bond. In typical 2-Cys Prxs, the C_R_–C_P_ disulfide connects antiparallel dimers, whereas in atypical 2-Cys Prxs, it forms intramolecularly. In order to complete the catalytic cycle, these disulfide bonds are reduced by a thioredoxin-type oxidoreductase [10–12]. In contrast, 1-Cys Prxs (such as human PrxVI) lack a C_R_ and instead form a mixed disulfide heterodimer with *π* glutathione S-transferase, which catalyzes the glutathione-driven reductive regeneration of the Prx [13, 14]. 

A remarkable feature of Prxs is their susceptibility to oxidative inactivation. Thus, C_P_ sulfenic acid can react with a second molecule of H_2_O_2_, which gives rise to C_P_ sulfinic acid. This leads to Prx inactivation, stabilization of decameric over dimeric configuration, and, in some cases, to an increase in chaperone activity [15–17]. At least in cytoplasmic and mitochondrial typical 2-Cys Prxs, sulfinic acid formation can be reversed by the action of sulfiredoxin at the expense of ATP [18, 19]. Under highly oxidizing conditions, C_P_ sulfinic acid can further and irreversibly react with a third molecule of H_2_O_2_ to form C_P_ sulfonic acid [15]. 

The GPx family is phylogenetically unrelated to Prxs but shares the ability to reduce hydroperoxide substrates [2]. A total of eight mammalian GPxs are known. They are subclassified into two groups according to the amino acid tetrad in their catalytic center. In SecGPxs (human GPx1–4 and 6) or CysGPxs (GPx5, 7, and 8), the common constituents Gln, Trp, and Asn are supplemented with a peroxidatic selenocysteine (Sec) or Cys, respectively [20]. Furthermore, GPxs differ with regard to their oligomeric state, with GPx1–3, 5, and 6 constituting homotetramers and GPx4, 7, and 8 monomers [21]. 

Upon hydroperoxide-mediated oxidation of the active-site selenocysteine, SecGPxs typically react with two molecules of glutathione (GSH) yielding glutathione disulfide (GSSG), which historically accounted for the generalized family name glutathione peroxidases [2, 21]. However, the use of GSH as reductant is not a common feature of GPxs nor is it strictly conserved within the SecGPx subgroup [2, 21–25]. In invertebrates and plants, monomeric CysGPxs harbor a C_R_ and exhibit an identical reaction mechanism as atypical 2-Cys Prxs (see above) [20, 26, 27]. In contrast, no typical C_R_ is present in the human monomeric CysGPxs GPx7 and 8. 

The ER serves many distinct cellular functions [28]. One of these is chaperone-mediated folding of nascent polypeptide chains, which often involves the introduction of disulfide bonds via oxidation of two adjacent cysteines. This process termed oxidative protein folding is driven by a number of distinct pathways, the most conserved of which involves the sulfhydryl oxidase endoplasmic oxidoreductin 1 (Ero1) as disulfide donor [29]. Since Ero1 can utilize molecular oxygen (O_2_) as terminal electron acceptor, it generates stoichiometric amounts of H_2_O_2_ for every disulfide bond produced, as demonstrated *in vitro* [30]. In addition, H_2_O_2_ sources other than the paralogs Ero1*α* and Ero1*β* exist within the mammalian ER. Although initially assigned to phagocytic cells only, more recent findings have shown that NOX family members are expressed in various cell types [3] where they produce H_2_O_2_ at different subcellular sites including the ER [31–33]. Likewise, the secreted quiescinsulfhydryl oxidases were identified as producers of H_2_O_2_ [34], although these enzymes function in the extracellular space [35] and their contribution to intracellular oxidative protein folding is uncertain [36, 37]. It has also been suggested that ROS produced by mitochondrial respiration could impact on disulfide-bond formation in secretory compartments including the ER [38]. Leakage of the mitochondrial electron transport chain, predominantly at complex III, releases superoxide and H_2_O_2_ into the intermembrane space of mitochondria [39, 40]. The close apposition of ER and mitochondria [41] could enable these ROS to contribute to ER-associated oxidative protein folding. 

This review will focus on PrxIV, GPx7, and GPx8, which reside in the ER of vertebrates, lancelets, ascidians, and—in case of PrxIV—echinoderms and arthropods [42]. As detailed further below, all ER-resident peroxidases can use protein disulfide isomerases (PDIs; the “thioredoxins of the ER”) as reducing substrates, allowing them to exploit the oxidizing power of ER peroxide sources for oxidative protein folding. However, reducing substrates other than PDIs may also participate in the reaction cycle of ER peroxidases. 

## 2. H_2_O_2_ in the ER: Bulk Metabolite or Locally Restricted Messenger?

**Figure 1:**
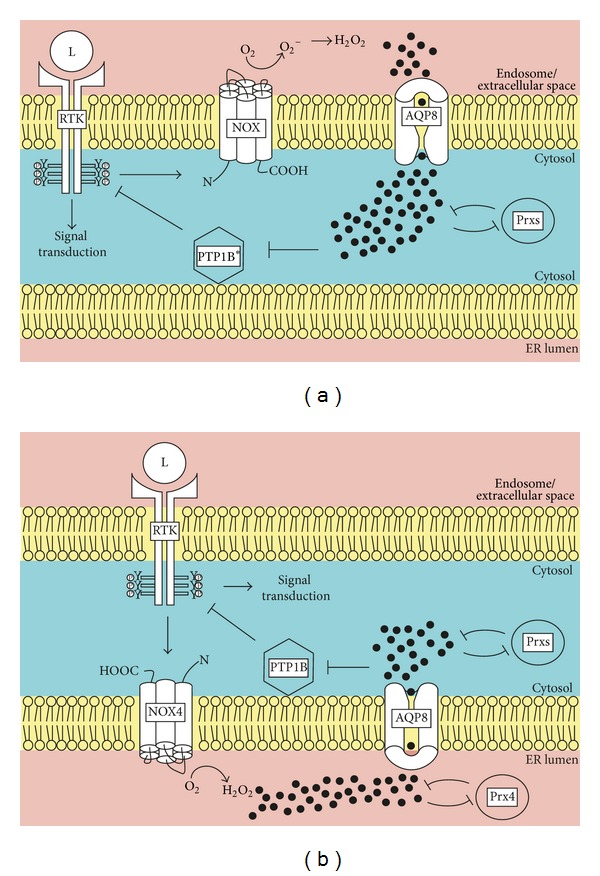
RTK signaling involves NOX-derived H_2_O_2_ as second messenger. (a) Binding of ligand (L) to receptor tyrosine kinases (RTK) on the cell surface activates NADPH oxidases (NOX) and leads to the generation of extracellular or, following endocytosis, endosomal superoxide (O_2_^−^), which can be dismutated to H_2_O_2_  (black filled circles). Upon aquaporin 8 (AQP8)-facilitated diffusion across the plasma/endosomal membrane, H_2_O_2_ locally inactivates the intracellular negative regulators phosphotyrosine phosphatases (PTPs) and peroxiredoxins (Prxs), which prolongs RTK signal transduction. This step mostly, but not exclusively (as depicted by an asterisk), involves the endoplasmic reticulum (ER)-associated PTP1B. Spatial restriction of H_2_O_2_ is achieved by cytosolic ROS scavengers like Prxs. (b) An ER-centered route of RTK-mediated signal transduction involves NOX4 in the ER membrane and PTP1B. In this context, ER-luminal buildup of H_2_O_2_ is controlled by ER-resident PrxIV.

Reliable detection of the cellular distribution of H_2_O_2_ is a challenging task. The recent development of genetically encoded sensors, which can be expressed in different subcellular compartments, significantly facilitated the monitoring of spatial and temporal changes in H_2_O_2_/ROS concentration [43]. For instance, targeted expression of the yellow fluorescent protein-based, ratiometric, and H_2_O_2_-sensitive HyPer sensor was used to record the oxidizing environment in the mammalian ER [33, 44–46]. On the basis of the predominantly oxidized state of ER-localized HyPer (HyPer_ER_) and the predominantly reduced state of HyPer on the cytoplasmic surface of the ER, a high [H_2_O_2_]_ER_, which is strictly confined to the lumen of the organelle, has been inferred [44]. Several lines of evidence argue against this interpretation though. First, as detailed in the following paragraph, numerous examples for signaling roles of ER-derived H_2_O_2_ are known, which suggest analogy to the critical involvement of Nox-derived H_2_O_2_ in receptor tyrosine kinase (RTK) signal transduction at the cell surface [47–50] (Figure 1). Second, the presence of peroxidases in the ER lumen (see below) appears incompatible with a high steady-state [H_2_O_2_]_ER_. Third, the demonstration of aquaporin 8-facilitated entry of H_2_O_2_ into the ER [8] suggests that aquaporin 8 can also facilitate exit of ER-derived H_2_O_2_ (see also Figure 1). Forth, since the ratiometric readout of HyPer is based on the formation of an intramolecular disulfide bond [51], oxidation of HyPer in the ER could be catalyzed by resident oxidoreductases independently of H_2_O_2_. Consistent with this assumption, no effect on HyPer_ER_ oxidation was observed upon overexpression of PrxIV or of ER-targeted catalase in pancreatic beta-cells [46]. The increased oxidation of HyPer_ER_ observed in response to higher levels of Ero1*α* [44, 52] can therefore reflect both enhanced oxidation of PDIs and a rise in [H_2_O_2_]_ER_. Thus, the Ero1*α*-induced increase in oxidation of HyPer_ER_ can only be partially reversed by addition of the H_2_O_2_ scavenger butylated hydroxyanisole (our unpublished observations). Conversely, increased oxidation of HyPer_ER_ in response to NOX4 induction is blunted by coexpression of catalase in the ER [33]. 

The role of H_2_O_2_ as signaling molecule typically manifests in the formation of short-lived microdomains of elevated [H_2_O_2_] [49, 53]. For instance, ligand binding to RTKs at the cell surface such as platelet-derived growth factor receptor, epidermal growth factor receptor (EGFR), or insulin receptor stimulates the local production of H_2_O_2_ via crosstalk with NOX enzymes [47, 49, 54, 55]. This leads to oxidative inactivation of protein tyrosine phosphatases (PTPs), which prolongs RTK signaling until cytosolic ROS scavengers such as Prxs have cleared H_2_O_2_ [56–60] (Figure 1(a)). At least in certain contexts, such H_2_O_2_-dependent signal amplification is mediated by ER-resident NOX4 and PTP1B [31] (Figure 1(b)). Thus, activated EGFR is internalized into endosomes and transported close to the ER [61] where its PTP1B-dependent dephosphorylation is negatively regulated by NOX4-derived H_2_O_2_ [31]. In the case of the granulocyte-colony stimulating factor receptor pathway, also ER-resident PrxIV (see next section) can modulate the signaling amplitude [62] (Figure 1(b)). 

NOX4-initiated signal transduction is linked to the adaptive/apoptotic output of the ER stress response—a conglomeration of ER-derived signaling cascades known as the unfolded protein response (UPR) [63]. In the context of atherosclerosis, oxysterol-stimulated smooth muscle cell apoptosis depends on NOX4, which is upregulated through the ER stress sensor Ire1*α* to produce H_2_O_2_ [32]. Similarly, NOX4 is induced in endothelial cells in response to a subset of ER stressors, leading to presumably locally restricted H_2_O_2_ signaling [33]. In both cases, proper activation of UPR pathways requires NOX4-derived H_2_O_2_. Of note, NOX4-dependent, ER-associated oxidative signaling through the RAS-ERK pathway in endothelial cells promotes prosurvival autophagy rather than cell death [33]. A related link operates in smooth muscle cells where NOX4-derived H_2_O_2_ stimulates autophagy by inhibiting autophagy-related gene 4B activity, which antagonizes ER stress and cell death [64].

Little is known about signaling roles of H_2_O_2_ sources other than NOX4 in the ER. Nevertheless, the available data on NOX4 strongly suggest that—in analogy to the situation in other compartments—H_2_O_2_ operates in the ER as a spatially restricted second messenger rather than a bulk metabolite. 

## 3. Peroxiredoxin IV

PrxIV is the only ER-resident representative of the Prx family. Its predominant isoform harbors a classical signal peptide, which is cleaved upon cotranslational entry into the ER, but no ER retrieval motif to ensure its retention in the early secretory pathway (ESP) [65, 66]. Instead, similar to the ER retention mechanism of Ero1*α*, physical interactions with the ESP oxidoreductases ERp44 and PDI inhibit PrxIV secretion from cells [67]. Therefore, cell-specific differences and/or saturation of the retrieval machinery, for example, following exogenous overexpression, might explain the ambiguity in the literature on the intracellular or secreted nature of PrxIV [68–72]. This review will focus on the role of the ER-resident fraction of PrxIV.

**Figure 2:**
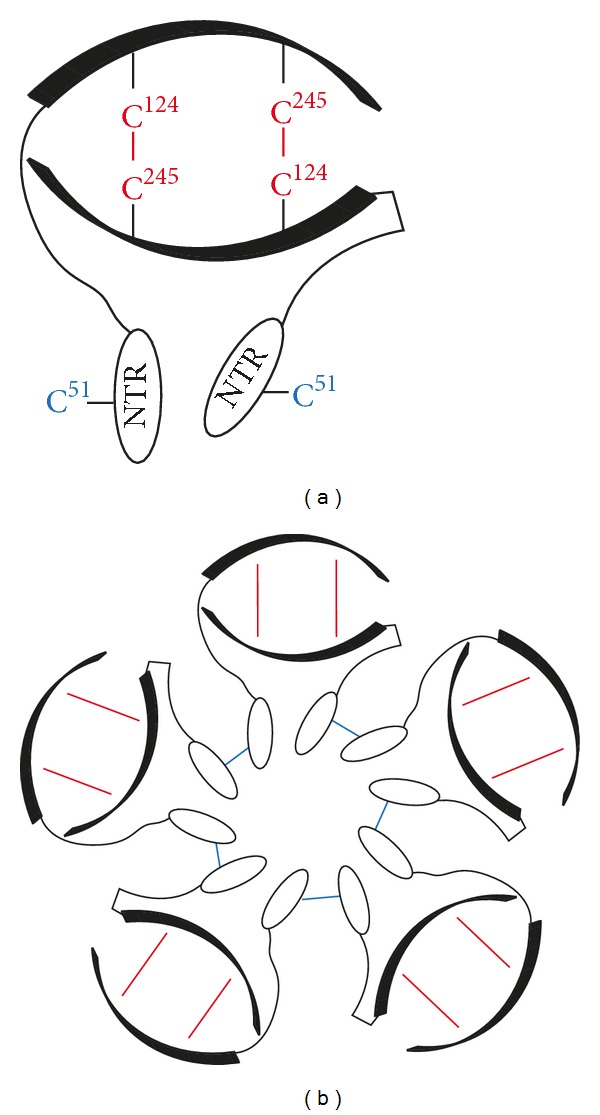
Oligomeric structure of PrxIV. (a) Upon peroxide-mediated oxidation, antiparallel PrxIV dimers are transiently linked by disulfide bonds between C_P_ (C^124^) on one subunit and C_R_ (C^245^) on the other subunit (depicted in red), which is the characteristic feature of typical 2-Cys Prxs. However, dimer formation relies on hydrophobic interactions and is redox state-independent. The flexible N-terminal region (NTR) of PrxIV is oriented towards the center of the toroid-shaped, decameric complex (b). The role of the disulfide bonds linking adjacent dimers via Cys^51^ in the NTR (depicted in blue) is currently unclear.

PrxIV belongs to the subclass of typical 2-Cys Prxs and predominantly exists in decameric configuration. The toroid shaped pentamer of antiparallel dimers (Figure 2) is stabilized by hydrophobic interactions at dimer-dimer interfaces. In contrast to other family members [73], PrxIV does not show significant transition from the decameric to the dimeric state upon disulfide-bond formation between C_P_ and C_R_, even though this process is associated with local unfolding [74]. Furthermore, PrxIV harbors a unique N-terminal extension. As judged from the positions of the truncated N-termini in the crystal structure, these flexible extensions protrude into the center of the decameric assembly of full length PrxIV protomers (Figure 2). In addition to hydrophobic interactions, neighboring antiparallel dimers are linked by Cys^51^–Cys^51^ interchain disulfide bonds between N-terminal regions (Figure 2), but mutagenesis to serine or alanine neither affected decamerization nor the catalytic parameters of PrxIV [74–76]. The impact of the N-terminal extensions for correct quaternary structure is still unclear. In an N-terminal truncation mutant, Wang et al. observed a significant transition from the decameric to the dimeric state upon oxidation. In contrast to this, Ikeda et al. reported a shift from decameric to higher oligomeric forms [76, 77]. 

Like other Prxs, PrxIV exhibits an exceptionally fast reactivity towards H_2_O_2_ (2.2 × 10^7^ M^−1^ s^−1^) [76]. As data on PrxIV reacting with peroxide substrates other than H_2_O_2_ is scarce, PrxIV may exclusively react with H_2_O_2_ *in vivo* (Table 1). PrxIV knockout cells stained with H_2_O_2_-reactive dye showed a bright signal, which was blunted upon reconstitution of PrxIV (Figure  S(10) in [62]). Where does this H_2_O_2_ come from? A popular model implicates Ero1*α*-derived H_2_O_2_, a regular byproduct of oxidative protein folding [78], as oxidizing substrate of PrxIV [79]. This model is based on the finding that activation of Ero1*α* in cells by dithiothreitol (DTT)-mediated reduction of its regulatory disulfide bonds increased the hyperoxidized fraction of PrxIV [80]. In further support, DTT-triggered hyperoxidation of PrxIV was inhibited by knockdown of Ero1*α* (Neil Bulleid, personal communication), and Ero1*α*-dependent accumulation of H_2_O_2_ in response to DTT treatment was increased by PrxIV knockdown and decreased by PrxIV overexpression (our unpublished observations). However, in contrast to GPx8 (see below), this crosstalk between Ero1*α*-derived H_2_O_2_ and PrxIV was only observed in the presence of DTT (our unpublished observations), which likely does not reflect normal physiology. Experiments with murine or fungal loss-of-function models of Ero1 strongly suggested that PrxIV can be coupled to (an) Ero1-independent source(s) of H_2_O_2_: ectopic expression of PrxIV rescues the thermosensitive *ero1-1* yeast strain by Ero1-independent oxidative protein folding [81] (see below) and PrxIV is required to protect Ero1-deficient mice against H_2_O_2_-mediated ascorbate depletion [82]. The H_2_O_2_ source(s) targeted by PrxIV remain(s) to be identified [12]. 

Following disulfide-bond formation between C_P_ and C_R_, PrxIV acts as PDI peroxidase by using several different PDIs as electron donors [75, 83] (Table 1). As discussed further below, these PDIs can subsequently shuttle the disulfide onto various substrate proteins, implicating PrxIV as an important element of oxidative protein folding. 

It is intriguing that despite the fact that the ER is devoid of sulfiredoxin activity, PrxIV has retained specific structural features to support H_2_O_2_-mediated hyperoxidation [74, 76]. Accordingly, sulfinylation of C_P_ in PrxIV could potentially serve a specific function. It has been speculated that hyperoxidized PrxIV could operate as a molecular chaperone or as a secreted damage associated molecular pattern [65]. 

## 4. GPx7 and GPx8

GPx7 and 8 are closely related ER-luminal members of the GPx family. Whereas GPx7 possesses a cleavable N-terminal signal sequence, GPx8 is a transmembrane protein with a short N-terminal cytoplasmic tail. Retention in the ESP is mediated by exposed, C-terminal motifs, -Arg-Glu-Asp-Leu and-Lys-Glu-Asp-Leu in GPx7 and 8, respectively, which are recognized in the Golgi by KDEL retrieval receptors [84]. This ESP-retention mechanism is noteworthy for GPx8, since ER membrane proteins are usually retrieved to the ER via cytosolic interactions with retrograde coat proteins [85]. The physiological implications of this peculiarity are currently unclear. 

**Figure 3:**
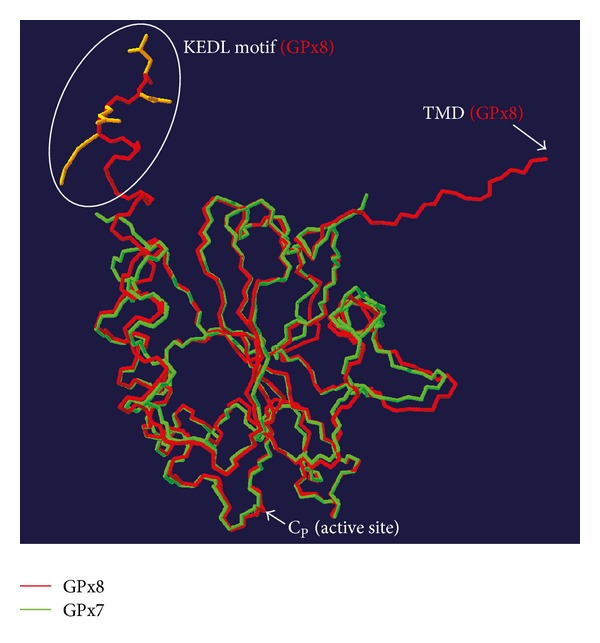
Superimposition of GPx7 and GPx8. Overlay of the carbon-nitrogen backbones of GPx7 (green; PDB ID 2KIJ) and GPx8 (red; PDB ID 2P31) was done using the Swiss PDB viewer software (available at http://www.expasy.org/). The close resemblance of the two three-dimensional structures is particularly appreciable in the peptide loops surrounding the active site Cys (C_P_). The ESP retention signal (KEDL motif) and the location of the transmembrane domain (TMD) of GPx8 (not part of the crystal structure) are indicated.

Whereas no other peroxide substrate besides H_2_O_2_ has been documented for GPx8 yet, GPx7 (also known as nonselenocysteine containing phospholipid hydroperoxide glutathione peroxidase, NPGPx) can efficiently react with phospholipid hydroperoxides *in vitro* (*k* > 10^3^ M^−1^ s^−1^, Table 1) [86]. Although speculative at present, we consider it possible that also in its native context, GPx7 can reduce lipid peroxidation products in the luminal leaflet of the ER membrane. As to GPx8, which largely shares the active site architecture with GPx7 (Figure 3), the short linker between the transmembrane anchor and the catalytic domain might not confer enough flexibility for the active site to interact with the lipid bilayer. Accordingly, both GPxs (together with PrxIV) could protect ER-oriented lipids against peroxidation by scavenging ER-luminal H_2_O_2_, but only soluble GPx7, in analogy to GPx4 [87], would be able to directly reverse lipid peroxidation by enzymatic reduction. 

Another prevailing model implicates Ero1 activity to provide H_2_O_2_ as oxidizing substrate for GPx7 and 8 [21, 88]. Using a split YFP complementation approach, Ero1*α* and GPx7 or 8 were found to associate within the ER, and addition of GPx7 increased the oxidase activity of Ero1*α* *in vitro* [88]. While the mechanistic basis for the latter finding remains to be elucidated, these data point to a functional interaction between GPxs and Ero1*α*. In line with this, knockdown of GPx8 but not PrxIV aggravated the accumulation of H_2_O_2_ induced by a deregulated Ero1*α* mutant (our unpublished observations). Therefore, despite their lower reactivity towards peroxide, the physical interaction with Ero1*α* likely places the GPxs in a privileged position relative to PrxIV to detoxify Ero1*α*-derived H_2_O_2_.

**Figure 4:**
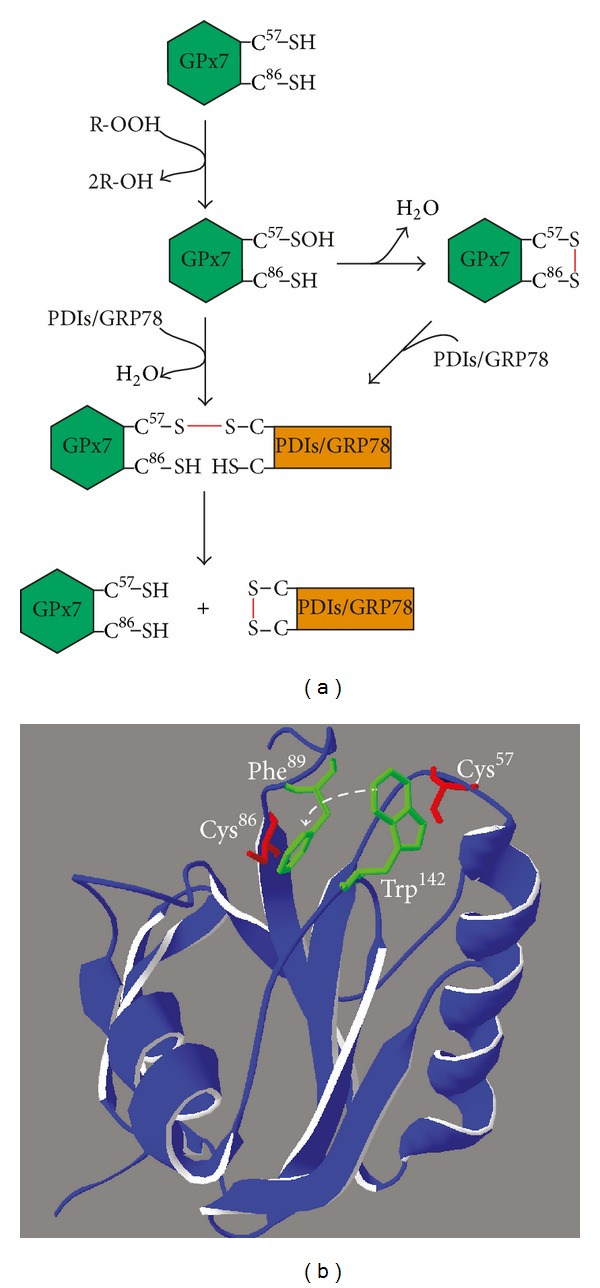
Suggested reaction mechanisms of GPx7. (a) Following peroxide-mediated oxidation of the active site Cys (C^57^), sulfenylated C^57^ is either directly subjected to nucleophilic attack by a (deprotonated) Cys in the reducing substrate (PDIs/GRP78) or attacked by (deprotonated) Cys^86^, which results in formation of an intramolecular disulfide bond. In a second step, this intramolecular disulfide is attacked by a Cys in the reducing substrate. Both pathways converge in the formation of an intermolecular disulfide-bonded intermediate between GPx7 and the reducing substrate prior to the completion of the reaction cycle, which gives rise to regenerated, reduced GPx7 and oxidized PDIs/GRP78. (b) Hypothesized conformational change prior to formation of a Cys^57^–Cys^86^ disulfide bond in GPx7 is depicted on the structure of reduced GPx7 (PDB ID 2KIJ). Active site rearrangement upon oxidation of Cys^57^ might involve a stacking interaction between the conserved aromatic side chains of Phe^89^ and Trp^142^ (green), which would move away Trp^142^ from Cys^57^ (dashed white arrow).

Irrespective of the peroxide source, the catalytic mechanism for the reductive regeneration of GPx7/8 remains controversial. Despite the absence of a canonical C_R_, GPx7 and 8 harbor an additional cysteine in a conserved Pro-Cys^86/108^-Asn-Gln-Phe motif [86]. Studies with GPx7 have highlighted two possible mechanisms of peroxidase reduction [86, 89, 90] (Figure 4(a)). Of note, one of the possibilities features Cys^86^ as a noncanonical C_R_. However, since C_P_ and Cys^86^ are ~11 Å apart in the crystal structure (Figure 4(b)), this implies a major conformational change. Indeed upon H_2_O_2_ addition, the intrinsic fluorescence of Trp^142^, which, in reduced GPx7, is particularly solvent-exposed and in close proximity to C_P_ (Figure 4(b)), readily resumes in the time scale of 2-3 sec after initial decline [88, 89]. This likely indicates the translocation of Trp^142^ away from the fluorescence-quenching C_P_ sulfenic acid. In this connection, we note the adjacent aromatic side chain of Phe^89^, which is part of the conserved motif surrounding Cys^86^ (see above), and speculate that stacking of Phe^89^ and Trp^142^ upon C_P_ oxidation could promote formation of the C_P_–Cys^86^ disulfide (Figure 4(b)). Interestingly, in addition to the Pro-Cys-Asn-Gln-Phe motif, the exposed Trp residue is conserved throughout the GPx family [86]. 

If GPx7 (and likely GPx8) can oxidize reducing substrates in the absence of Cys^86/108^, what could be the reason for its conservation? We suggest that the function of C_R_-dependent intramolecular disulfide-bond formation is to prevent the accumulation of sulfenylated GPxs, which may display reactivity towards nonnative thiol substrates. Rapid reaction with Cys^86^ largely prevents the accumulation of the C_P_-sulfenylated form of purified GPx7 in presence of H_2_O_2_ [89]. It will be interesting to assay the oxidation state of GPx7 and 8 in living cells. At all events, evidence for a possible toxic gain-of-function of sulfenylated GPxs came from experiments with an engineered H_2_O_2_-sensing fluorescent protein [91]. This protein is a fusion of redox-sensitive GFP (roGFP2) and Orp1, which is yeast GPx3. Mutation of C_R_ in Orp1 accelerated disulfide-bond formation in roGFP2 in response to H_2_O_2_ *in vitro*. In living cells, however, the C_R_-mutant sensor failed to respond to H_2_O_2_ addition, which was due to competing reactions with reducing substrates other than roGFP2 including glutathione [91]. 

## 5. Reducing Substrates of ER-Resident GPxs

In analogy to PrxIV, oxidized GPx7 and 8 were demonstrated to act as PDI peroxidases by using several different PDIs as electron donors [88] (Table 1). The utility of disulfide transfer onto PDIs shall be discussed in the next section. Here, we will touch upon alternative reducing substrates, which have been found to interact with GPx7 (Table 1). For instance, although glutathione reduces sulfenylated GPx7 at a far lower rate compared to PDI, it has been calculated to potentially represent a competing substrate taking into account its millimolar concentration *in vivo* [86]. However, since the reaction of glutathione with oxidized PDI is very fast [92], the physiological relevance of direct glutathione-mediated reduction of GPx7 is questionable. 

In contrast, disulfide transfer from GPx7 to the abundant ER chaperone and UPR target GRP78/BiP—as evidenced by cysteine-dependent coimmunoprecipitation from H_2_O_2_-treated cells—appears to have critical influence on ER physiology [90]. GRP78/BiP carrying the resulting Cys^41^–Cys^420^ disulfide exhibits increased chaperone activity towards misfolded clients, arguing for a role of GPx7 as oxidative stress sensor and positive regulator of GRP78/BiP [90]. Consistently, cells lacking active GPx7 were more susceptible to H_2_O_2_ and ER-stress-induced toxicity than wild-type control cells [90]. Very much like PrxIV knockout cells (see above), they also displayed increased staining with a H_2_O_2_-reactive dye compared to wild-type [90]. 

Nontargeting siRNA-transfected GPx7 knockout cells displayed harmfully elevated levels of siRNA compared to transfected wild-type cells, indicating a potential link between ER-resident GPx7 and the degradation machinery of nontargeting cytoplasmic siRNA [93]. This link was proposed to involve thiol-disulfide transfer between GPx7 and the nuclear exoribonuclease XRN2, although this reaction appears topologically prohibited [93]. Irrespective of this paradox but consistent with a role of GPx7 in the processing of small RNAs, nontargeting siRNA selectively induced GPx7 expression in wild-type fibroblasts [93], a process mediated by the nuclear protein nucleolin and its activity as transactivator of the GPx7 promotor [94]. It is interesting to note that the cytosolic membrane leaflet of the rough ER is emerging as a central nucleation site of miRNA/siRNA processing in plants and animals [95, 96], and the interplay between the RNA silencing machinery and GPx7 (and possibly other ER-resident peroxidases) deserves further attention. 

Compared to GPx7, the enzymatic characterization of GPx8 including the identification of its reducing substrates is far less developed. However, since the structures of their active sites are nearly superimposable (Figure 3), GPx7 and 8 are likely to share many of their catalytic properties. 

## 6. The Two-Disulfides-out-of-One-O_2_ Concept

**Figure 5:**
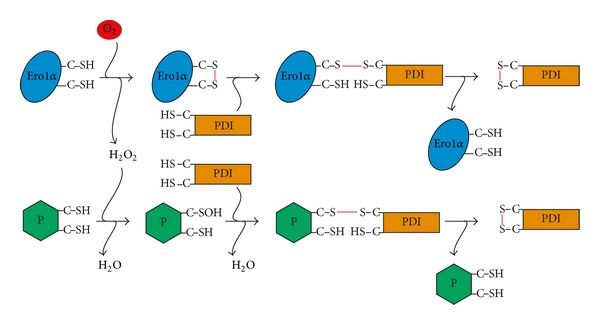
The two-disulfides-out-of-one-O_2_ concept. O_2_ (red)-mediated oxidation of Ero1*α* results in the generation of one disulfide bond (red), which is transferred to a reduced PDI, and of one molecule of H_2_O_2_. ER-resident peroxidases (P)—probably exclusively of the GPx family (see main text for details)—can couple the reduction of Ero1*α*-derived H_2_O_2_ to H_2_O with the introduction of a second disulfide bond (red) into a PDI family member, thereby exploiting the oxidizing capacity of H_2_O_2_.

Oxidative protein folding relies on *de novo* disulfide generating enzymes and on oxidants, which accept the electrons derived from thiol oxidation. While several such electron transfer cascades exist in the mammalian ER, resulting in a certain degree of redundancy, Ero1 oxidases (using O_2_ as oxidant) and PrxIV (using H_2_O_2_ as oxidant) are evidently the dominant disulfide sources [29, 36, 81]. The fact that both enzymes can oxidize PDIs [75, 78, 81, 83, 97, 98] has led to the intriguing concept that the four oxidizing equivalents in O_2_ can be exploited by the consecutive activity of Ero1 and PrxIV to generate two disulfides for oxidative protein folding [79, 99] (Figure 5). Along the same lines, the PDI peroxidase activity of GPx7 constitutes a pathway for the productive use of Ero1*α*-derived H_2_O_2_ in the biosynthesis of disulfides [88, 89]. 

Evidence for a contribution of ER-resident peroxidases to oxidative protein folding is manifold. Mixed disulfide reaction intermediates between peroxidase and PDI were isolated from cells [75, 81, 89], and in the case of PrxIV, interactions with the PDI family members ERp46 and P5 were also reported [75, 83]. Interestingly, of the two Cys-X-X-Cys active sites in PDI, PrxIV preferentially oxidizes the **a**′ domain active site and GPx7 the **a** domain active site [75, 89]. Since the mixed-disulfide complexes were stabilized by a Cys-X-X-Ala active site configuration in PDI [75], they must have resulted from the reaction of reduced PDI with oxidized peroxidase [100]. Accordingly, consumed peroxidase molecules can be activated/recycled by PDIs. It is possible that the availability of reduced PDIs actively adjusts the activation state of ER peroxidases. Thus, peroxidases could be kept in an inactive state unless new disulfides are needed, as indicated by the accumulation of reduced PDIs. In a very related manner, the intramolecular disulfides, which shut off Ero1*α*, are feedback-regulated by the availability of reduced PDI [101]. In contrast to Ero1*α*, however, the redox state of PrxIV appears to be predominantly reduced in cells at steady state [83]. 

Peroxidase/PDI-catalyzed oxidative protein folding can be reconstituted. Refolding of reduced RNase A, a process requiring introduction of four disulfides, occurs in the presence of PDI together with PrxIV or GPx7 [81, 89]. It is important to note though that PrxIV-driven refolding appears to depend on the addition of H_2_O_2_, whereas GPx7-driven refolding readily works in presence of Ero1*α*, which generates H_2_O_2_ by reducing ambient O_2_ [81, 89]. This difference parallels the evidence discussed above for a preference of GPx7 or 8 over PrxIV to detoxify Ero1*α*-derived H_2_O_2_. 

The role of PrxIV as a source of disulfide bonds is also strongly supported by genetics. Ero1-deficient mouse embryonic fibroblasts are hypersensitive to the loss of PrxIV, which causes hypooxidation of an ER-targeted thiol-disulfide sensor, ER dilation, and decreased cell viability [81]. Somewhat counterintuitively, compound loss of Ero1*α*/*β* and PrxIV also leads to oxidative phenotypes such as glutathione depletion and cell senescence [82]. These phenotypes are attributed to the failure to reduce H_2_O_2_ from as yet unidentified origin, which causes shortage of intracellular ascorbate (vitamin C) associated with defects in collagen synthesis and scurvy [82]. Last but not least, codepletion of PrxIV in hepatocytes exacerbates the cytotoxic phenotype of Ero1*α*/*β* depletion and further slows ER reoxidation after reductive challenge [36]. 

Taken together, a role in oxidative protein folding is particularly well documented for PrxIV but is also shared by the ER-resident GPxs. Still, although appealing, we consider it likely that the concept of peroxidase-dependent exploitation of Ero1*α*-derived H_2_O_2_ (Figure 5) only applies to GPxs (see above). 

## 7. Organismal Roles of ER Peroxidases

For PrxIV and GPx7, *in vivo* studies have been performed in different model organisms. One striking conclusion of these studies is that whole-body loss-of-function of GPx7 in mice shows a stronger organismal phenotype compared to PrxIV deficiency. No *in vivo* characterization of the role of GPx8 has been published so far. 

Male mice lacking a functional X-chromosomal *PRDX4* gene (PrxIV^−/y^) display a mild phenotype, which manifests predominantly by testicular atrophy accompanied by increased DNA fragmentation and peroxidation of lipids and proteins [69]. The number of sperms is markedly decreased in the epididymis of PrxIV^−/y^ mice, which, however, does not affect their fertility [69]. These phenotypes are likely attributed to loss of the testis-specific transmembrane isoform of PrxIV [65]. 

Similarly, in fruit flies a decrease in PrxIV expression to 10–20% of wild-type levels is associated with increased [H_2_O_2_] and lipid peroxidation in membrane preparations from whole animals [102]. However, negative impact on longevity was only observed under oxidative stress conditions induced by H_2_O_2_ or paraquat treatment. Strikingly, 6–10 fold, global overexpression of PrxIV in flies, which shifted its subcellular distribution from predominantly ER-resident to cytosolic and secreted, resulted in dramatically shortened lifespan under nonstress conditions and increased apoptosis in thoracic muscle and fat body tissue [102]. Since this proapoptotic phenotype upon PrxIV overexpression was not reproducible in cultured fly cells, noncell autonomous and/or fly-specific *in vivo* effects of secreted PrxIV need further consideration. 

In contrast to this, overexpression of PrxIV in mice has beneficial effects in the context of metabolic diseases. For instance, elevated levels of PrxIV in apolipoprotein E negative mice, which were fed a high cholesterol diet, have antiatherogenic effects with less oxidative stress, a decrease in apoptosis, and suppressed T-lymphocyte infiltration [103]. In addition, cytoprotective effects of overexpressed PrxIV were evident in nongenetic mouse models of both type 1 and type 2 diabetes mellitus (T1DM and T2DM) [104, 105]. Specifically, autoimmune-induced apoptosis of pancreatic *β*-cells (in T1DM) and fatty liver phenotypes and peripheral insulin resistance (in T2DM) were diminished upon PrxIV overexpression. It is possible that more efficient clearance of inflammatory ROS is the underlying reason for the ameliorated phenotypes of these mice [104, 105]. However, one has to bear in mind that overexpression of PrxIV above a certain threshold exceeds ERp44-mediated ESP retrieval [67] and therefore may result in abnormally high levels of secreted peroxidase. Overexpression studies therefore need careful evaluation, before implications on normal physiology can be conclusively deduced. 

Interestingly, endogenous PrxIV is dramatically upregulated during terminal B-cell differentiation [106], a process accompanied by increased ROS levels but not by discernible hyperoxidation of the ER lumen [107, 108]. PrxIV knockout splenocytes, however, develop normally and do not show a defect in antibody secretion, arguing for redundancy among different oxidant control mechanisms [106]. 

In contrast to the relatively mild PrxIV knockout phenotype [69], quite dramatic changes including a shortened lifespan were documented for GPx7^−/−^ compared to control mice [90]. Besides induction of UPR hallmarks in different organs, these mice exhibited oxidative DNA damage and apoptosis predominantly in the kidney. Furthermore, multiple organ dysfunctions including glomerulonephritis, spleno- and cardiomegaly, fatty liver, and multiple malignant neoplasms were diagnosed [90]. Carcinogenesis and premature death were concluded to reflect systemic oxidative stress [90]. 

Along this line, Peng and coworkers proposed a tumor-suppressive role for GPx7 in oesophageal epithelial cells [109]. Progression from healthy tissue to premalignant Barrett's oesophagus (BO) and further to malignant oesophageal adenocarcinoma (OAC) is associated with gastro-oesophageal reflux, leading to ROS accumulation and increased oxidative DNA damage. BO/OAC neoplastic transformation is accompanied by decreased expression of GPx7 [110]. The diminished levels of GPx7 in BO and OAC tissues are due to DNA-hypermethylation within the respective promoter region. Bile acid-mediated intracellular and extracellular ROS accumulation in oesophageal epithelial cell culture was also responsive to overexpression or downregulation of GPx7 [111]. Furthermore, reconstitution of GPx7 expression suppressed growth and promoted cellular senescence in both *in vitro *and *in vivo* OAC models [109]. Therefore, inactivation of GPx7 is a crucial step in BO/OAC formation. Despite these conclusive links between oxidative injury and GPx7 expression *in vivo*, it is important to emphasize that the actual source of peroxide that causes ROS accumulation in absence of GPx7 remains to be identified. A possible involvement of Ero1*α* [112] remains to be experimentally verified. 

## 8. Conclusions and Perspectives

The reaction cycle of a peroxidase is split into an oxidizing part, which uses a source of hydroperoxide, and a reductive part, which uses a dithiol substrate. As such, available data highlight a twofold function of ER-resident peroxidases; on one hand, they can reduce and spatially restrict local H_2_O_2_ or lipid hydroperoxides and on the other hand, they are net producers of disulfide bonds. 

The model, which has probably generated the highest resonance, holds that ER peroxidases eliminate the obligatory and potentially harmful side product of Ero1-catalyzed disulfide-bond formation, H_2_O_2_, by exploiting its oxidizing power to generate a second disulfide in PDI for oxidative protein folding (Figure 5). The fact that all ER peroxidases—PrxIV, GPx7, and GPx8—can catalyze steps of this pathway *in vitro* [75, 81, 88, 89] has led to the understanding that they basically perform the same function [65]. But do ER peroxidases really all do the same? Are their functions redundant? We believe that this is clearly not the case. For instance, the prominent phenotype of the GPx7^−/−^ mouse strongly suggests that neither PrxIV nor GPx8 can broadly substitute for the loss of GPx7 [90]. This could be due to the fact that GPx7 uses unique reducing substrates (other than PDI family members) or metabolizes phospholipid hydroperoxides in the ER-facing membrane leaflet *in vivo*. Alternatively, tissue-specific expression levels might prohibit functional compensation between ER peroxidases. These questions are exciting subjects for future research. Clearly, it will also be interesting to learn about the phenotypes of GPx8^−/−^ and GPx7/8 double knockout animals. Whether or not other human GPx isoforms like for example, the ubiquitously secreted GPx3 [21] have an additional intracellular function in the ER is another open question. 

Differences between ER peroxidases also manifest in terms of the source of hydroperoxide. There is clear proof for PrxIV reacting with Ero1-independent H_2_O_2_ [81, 82], and unpublished data from our laboratory has demonstrated that this peroxidase does not react with Ero1*α*-derived H_2_O_2_ in cells under steady-state conditions. In this respect, one of the most urgent questions is which is the H_2_O_2_ source that drives PrxIV-dependent oxidative protein folding [36, 81, 82]. Identification of this source will likely provide major new insights into the diffusion pathways of this metabolite. 

Another area for future investigation concerns potential signaling roles of H_2_O_2_ in the ER lumen and beyond. For instance, the interplay of ER-resident NOX family members and peroxidases is largely unexplored. Likewise, it is currently unclear whether or not the known proapoptotic role of Ero1*α* during ER stress [113–115] is mediated by diffusion of Ero1*α*-derived H_2_O_2_ into the cytoplasm, as is suggested [7]. It is foreseeable that aquaporins will be found to play a central function in these processes at the ER membrane [8]. As every discovery arouses further interest and curiosity, we are expecting new insights and again new questions to come. 

## Figures and Tables

**Table 1:** Published peroxide and reducing substrates of ER-resident peroxidases.

	Peroxide substrates	Reducing substrates
PrxIV	H_2_O_2_ [76]	PDIs (ERp46, P5, PDI) [75, 83]
GPx7	H_2_O_2_ [88] phospholipid hydroperoxide [86]	PDIs (PDI, ERp46, ERp57, ERp72, P5) [86, 88, 89], GRP78/BiP [90], GSH [86], XRN2 [93]
GPx8	H_2_O_2_ [88]	PDIs (PDI, ERp46, ERp57, ERp72, P5) [88]
